# Phages for Biofilm Removal

**DOI:** 10.3390/antibiotics9050268

**Published:** 2020-05-21

**Authors:** Celia Ferriol-González, Pilar Domingo-Calap

**Affiliations:** 1 Department of Genetics, Universitat de València, 46100 Valencia, Spain; celia.ferriol@gmail.com; 2 Institute for Integrative Systems Biology, I^2^SysBio, Universitat de València-CSIC, 46910 Valencia, Spain

**Keywords:** biofilm, bacteriophage, phage therapy, antibiotic resistance

## Abstract

Biofilms are clusters of bacteria that live in association with surfaces. Their main characteristic is that the bacteria inside the biofilms are attached to other bacterial cells and to the surface by an extracellular polymeric matrix. Biofilms are capable of adhering to a wide variety of surfaces, both biotic and abiotic, including human tissues, medical devices, and other materials. On these surfaces, biofilms represent a major threat causing infectious diseases and economic losses. In addition, current antibiotics and common disinfectants have shown limited ability to remove biofilms adequately, and phage-based treatments are proposed as promising alternatives for biofilm eradication. This review analyzes the main advantages and challenges that phages can offer for the elimination of biofilms, as well as the most important factors to be taken into account in order to design effective phage-based treatments.

## 1. Introduction

**Figure 1:**
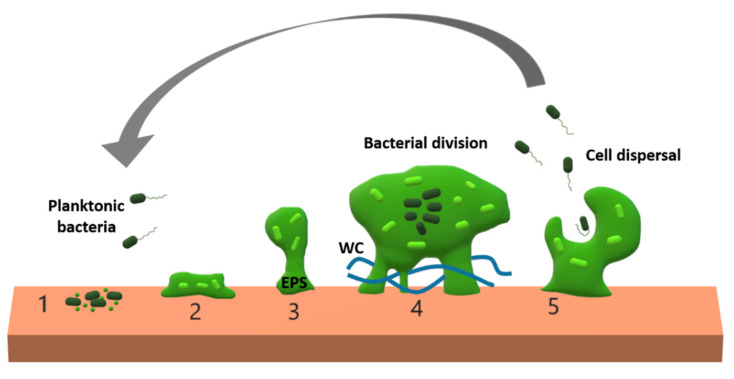
Schematic representation of biofilm formation. 1. Planktonic bacteria establish their initial adhesion to a surface. 2. The cells start to produce an extracellular polymeric substances (EPS) matrix and divide. 3. The bacterial population grows, increasing the bacterial density, and activating *quorum sensing* signaling-depending processes. 4. *Quorum sensing* regulates the development of specialized cells and division of labor. The biofilm matrix contains extracellular enzymes and water channels (WC), that facilitate access to nutrients. 5. Activation of biofilm disruption. Some cells can disperse and initiate new biofilms. Note that biofilms can be formed by multiple species of bacteria.

Although bacteria are commonly found in nature as individual cells, they can also form multicellular structures called biofilms [1]. Biofilms are complex clusters of bacteria, containing one or more species. They are bound by extracellular polymeric substances (EPS) and attached to surfaces such as living tissue, medical devices, food, industrial equipment, or pipes, among others [2,3,4,5]. This extracellular matrix is the immediate bacterial environment within the biofilm, produced predominantly by the bacteria themselves. The EPS matrix consists mainly of exopolysaccharides, but may also contain proteins, nucleic acids, and lipids. This polymeric network connects and immobilizes the cells within the biofilm, providing mechanical stability and adhesion to surfaces. Each biofilm has its own architecture, determined mainly by the matrix. In addition, an aqueous channel system connects the embedded cells, allowing them to access nutrients. The biofilm matrix also contains extracellular enzymes, which act as an external digestive system to help extract nutrients (Figure 1) [2,6,7].

Biofilm formation is a cooperative group behavior that begins with the adhesion of the first cells to a given surface. In this first step, cell motility can help bacteria to reach the surface, but is not essential to the process. The mechanisms of motility include flagella, fimbriae, and other surface proteins. Once adhered to the surface, cells begin to divide and the formation of the EPS matrix fixes the initial adhesion [7,8]. Coordination of the different bacteria within the biofilm is necessary for this and involves chemical communication between the cells. *Quorum sensing* is a mechanism of cell–cell communication, which consists of synchronizing gene expression in response to population cell density. The *quorum sensing* system allows bacteria to detect population density based on the accumulation of specific signaling molecules [9]. When population density is high, the accumulation of signals triggers different processes, modulating survival strategies through the differential expression of genes, including those involved in virulence [10,11,12]. Therefore, population density is an important determinant for coordinating the change to a biofilm lifestyle, or for activating the maturation of biofilm disruption. The signaling molecules involved can be from the same or different species [13]. Bacterial biofilms exhibit a division of labor, also related to *quorum sensing* and determination of the fate of the biofilm formation process [9,14]. Finally, in mature biofilms, some cells disperse, allowing the colonization of new surfaces and the formation of new biofilms [10,15].

The formation of bacterial biofilms is often considered a virulence factor [11]. Antibiotics are not suitable for removing biofilms, mainly because of the antibiotic tolerance of the bacteria within the biofilms. While drug resistance is often referred to as a genetic process resulting from spontaneous mutations or horizontal gene transfer, tolerance is a phenotypically defined process by which bacteria survive the effect of a particular antibiotic in a given environment [16]. Subsequent bacterial replication in the presence of antibiotics can promote the mutation and selection process necessary for the emergence of drug-resistant strains. Biofilms typically confer tolerance to antibiotics by providing a physical barrier, but also because innermost cells are less metabolically active and therefore less affected by antibiotics [17,18,19,20]. Stress responses can limit bacterial growth, especially oxygen depletion, forcing bacteria to use alternative metabolic pathways leading to increased antibiotic tolerance [20]. Biofilm cells thus exhibit physiological heterogeneity, as revealed by differences in gene expression, metabolic activity, and phenotypic characteristics of bacteria located in different areas of the biofilm.

Given the role that biofilms play in tolerance and resistance to antibiotics, new treatments aimed at eliminating them are needed, and bacteriophages could be an interesting alternative. However, the commercial use of phages is still incipient due to several concerns. On the one hand, there are no laws for their specific license, sale, and distribution. On the other hand, due to the lack of regulation of their use in humans, clinical trials are under-represented [21]. In addition to these challenges, social and political awareness hamper the development of phage-based products [22]. Nevertheless, phages show interesting properties in terms of biofilm removal, as they produce specific enzymes that allow them to actively penetrate and disrupt biofilms. Other advantages of phages include their multiplicity at the infection site, their high specificity, avoiding the appearance of side effects, and their evolving capacity. However, the large diversity of phages reveals the lack of characterization and misunderstanding of their genome content [23]. Another drawback of phages in their use as therapeutic tools in humans or animals is that they can provoke a rapid release of bacterial endotoxins, leading to undesirable inflammatory responses [24]. In this review, we summarize how phage/biofilm interactions work and discuss the use of phages to combat biofilm forming bacteria.

## 2. Biofilm Features Affecting Phage Penetration, Diffusion, and Propagation

In order to design effective methods for phage-based biofilm removal, it is important to understand basic mechanisms such as penetration, diffusion, and propagation of phages within biofilms. In general, these processes are determined by the structure of the biofilm matrix, the physiological heterogeneity within the biofilm, and the bacterial species and strains that form the biofilm [25].

### 2.1. Anti-Phage Role of Biofilm Matrices

The EPS matrix plays a natural defense role against phages [26,27,28,29]. Biofilm trapping of phage particles depends on the composition of the matrix and the bacterial surface. Such entrapment reduces the phage recognition of the bacterial receptors and therefore can efficiently prevent infection [25]. In addition, the biofilm matrix contains phage-inactivating enzymes secreted by the bacteria within the biofilm [6,30]. The age of the biofilm has also been shown to be an important parameter affecting the tolerance of the biofilm to phages, since during maturation of the biofilm the matrix becomes less favorable to phage diffusion. This can be a limitation for phage therapy, particularly for treating chronic bacterial infections. However, phage infection of less metabolically active bacteria may still produce new virions, although phages will prefer to exploit newly divided biofilm-surface bacteria. Thus, phage therapy will be partially effective, depending on the vulnerability to phages, requiring more aggressive or extensive treatment to eradicate biofilms [31]. In general, biofilm thickness is a good indicator of the ability of a biofilm to prevent phage infection [25,32]. On the other hand, it has been suggested that biofilms can sometimes function as phage deposits because the matrix offers protection from degradation to phages [32]. The structure of the water channel network within the biofilm is also important for the penetration and diffusion of phages, as they can easily spread through these channels [33].

### 2.2. Physiological Heterogeneity Inside Biofilms

Bacteria located in the superficial areas of the biofilm exhibit different physiological properties from those located in more internal regions. Bacteria on the surface of the biofilm are constantly renewed and are key to initiating phage infection. They are metabolically active and the EPS matrix is newly formed and therefore less structured. These characteristics can make the surface bacteria more vulnerable to phage infection [18,29,31]. In contrast, bacteria from deeper layers of the biofilm have restricted access to nutrients and oxygen, leading to considerably slower growth, but also reduced sensitivity to antibiotics and phages. Therefore, the depth of the biofilm determines both the availability of nutrients and the penetration, diffusion, and spread of phages [18,27]. The number of cells with reduced metabolic activity in biofilms may increase with the age of the biofilm. In addition, reduced metabolic activity decreases the lytic effect of phages reducing their biofilm-removal ability. Therefore, older biofilms (in particular older bacteria within biofilms) are more difficult to remove using phages than younger biofilms [31,34].

### 2.3. Species and Strain Composition of the Biofilm

Another important parameter affecting phage penetration is the type of bacterial strains and species that form the biofilm [35]. Biofilms in nature are usually multi-species systems. However, most studies on phage biofilm control focus on single-species biofilms. Phage interactions with multi-species biofilms is a complex process, since these biofilms have a higher polymer diversity and a heterogeneous spatial distribution of bacteria and their polymers, which may decrease phage penetration due to the specificity of the phage target and phage degradation enzymes [36,37]. For example, depending on the environmental conditions under which the biofilm is infected (static or dynamic conditions), the physical interactions between the biofilm and the phages may be affected, modifying the success of phage-based treatments [38]. The effect of phages on biofilms has been studied in communities of two species consisting of phage-resistant and phage-susceptible bacteria. In this system, relative fitness depends both on competition for resources and on the pressure exerted by the phage infection. The results obtained suggest that species composition of a biofilm may influence the success of phage-based therapies [39].

## 3. Bacteria–Phage Co-Evolution Within Biofilms

Phages and bacteria can often evolve together in antagonistic co-evolutionary cycles, accelerating the rate of evolution of several traits, including virulence and biofilm formation. The EPS matrix confers a physical barrier against phages, that apparently allows bacteria in the biofilm to develop specific phage resistance mechanisms not seen in free bacteria [40]. Interestingly, the ability of phages to co-evolve with their hosts allows them to escape the emergence of bacterial resistance mechanisms [41]. Several mechanisms have been described in bacteria to reduce phage infection, many of which are shared between biofilm and planktonic bacteria. For example, bacteria can prevent phage adsorption by blocking their surface receptor mechanisms, inactivating them or limiting their access [42,43,44,45]. Similarly, bacteria can protect themselves by producing an EPS capsule, which limits the access of external factors to the cells, including phages [26,27,28,29]. To overcome this barrier, many phages encode a variety of enzymes that allow their direct penetration into the biofilm. Another interesting mechanism to prevent infection of phages is to specifically recognize the nucleic acids of the phages and destroy them. Restriction-modification systems, and especially the bacterial immune system called regularly spaced short palindromic repeats (CRISPR) can prevent infection [46]. As a final barrier to phage infection, the bacteria can use an abortive infection system that leads to the death of the infected cell, preventing the spread of phages through the community [40,45]. However, phages have developed mechanisms to overcome these resistances.

*Quorum sensing* signals have also been linked to phage resistance in biofilms [12]. *Quorum sensing* can, for example, modulate the number of phage receptors on cell surfaces. This mechanism has been described in *Escherichia coli*, where, in response to the N-acyl-l-homoserine lactone, a reduction in the number of λ receptors on the bacterial surface is observed, which directly reduces the rate of phage adsorption [47]. *Quorum sensing* may also be involved in defense against phage infection by influencing the viable cell population and its physiological status. This has been observed in K5 *Pseudomonas aeruginosa* phage, where the presence of penicillin acid, a *quorum sensing* inhibitor, increased the efficiency of infection [48].

## 4. Phage Applications against Biofilms

**Figure 2:**
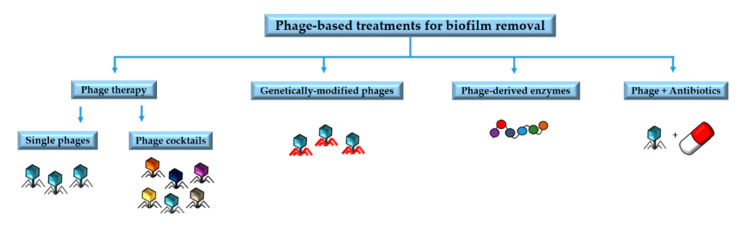
Main phage-based treatments for biofilm removal.

Understanding the underlying mechanisms involved in phage resistance and the co-evolutionary interactions between phages and biofilms is very important to design phage-based treatments and to minimize the likelihood of resistance emergence [49]. Phage-based treatments include phage therapy involving single phages or phage cocktails, phage-derived enzymes, phages in combination with antibiotics, and genetically modified phages [34]. In this section we will summarize some of the main applications of phages and their by-products for the removal of biofilms (Figure 2).

### 4.1. Phage Therapy

Since phages can actively penetrate and disturb biofilms in nature, they can be used to obtain specific and improved treatments against biofilms [50]. Phage-based therapies focus on lytic phages because they destroy their bacterial hosts, but also because they lack integrases and other enzymes involved in horizontal gene transfer [51]. In order to design phage-based methods to remove biofilms, it is important to take into account the specific characteristics of the phages that may play a role in their penetration, diffusion, and propagation through the biofilm. For example, penetration of the biofilm is often less efficient for larger phages [52].

Phages encoding EPS-degrading enzymes are of particular interest against biofilms. Depolymerases are enzymes encoded by phages that specifically degrade EPS matrix components, improving phage penetration [36]. Another source of EPS-degrading enzymes are the bacteria found inside the biofilm under stress conditions. Stress can be triggered by phage infection, facilitating increased penetration and dissemination of phages within the community. This has been demonstrated in *Pseudomonas aeruginosa* biofilms, where phage infection was found to reduce the viscosity of biofilms by bacterial enzymes [28]. Phage therapy against biofilms of *P. aeruginosa* has been also tested in a mouse model of cystic fibrosis and has been shown to successfully remove biofilms [53]. Phages have also been shown to be effective against oral biofilms that cause infections such as caries, periodontal and peri-implant disease, including *Enterococcus faecalis*, *Fusobacterium nucleatum*, and *Streptococcus* spp. among others, suggesting promising new oral health products based on phages [54].

Antibiotics are usually broad-spectrum stable chemical compounds, while phages are very specific and evolving entities. On the one hand, their specificity is an advantage, as it reduces off-target damage and restricts the development of resistance to target-specific bacteria [41]. In addition, phages are evolving entities that can counteract bacterial resistance. On the other hand, specificity is also a limitation because it requires great efforts in terms of phage bioprospecting. Furthermore, specificity means that the bacterial pathogen has to be identified at species or even strain level before treatment is administered, which can be a problem for acute infections requiring a rapid response. This issue can be addressed by phage cocktails. If the target is a single species or strain, phages that do not infect the target will simply function as a bystander. However, biofilms are often multi-species communities, which means that cocktails can contribute to disrupting biofilms more efficiently [55]. Another interesting aspect of phage cocktails is that they can prevent the emergence of phage resistant bacteria if multiple phages active against a given target are included in the cocktail [40,56]. In addition, the phages within a cocktail can interact synergistically, increasing lytic activity [57]. However, interference or antagonistic interactions between phages could be also possible.

Recent studies support the use of cocktails against bacterial biofilms in vivo, especially for multi-species biofilms. For example, a phage cocktail was formulated to treat catheter-associated urinary tract infections caused by *Proteus mirabilis*, showing strong biofilm destruction activity and preventing biofilm formation. The application of this cocktail in liquid or gel form to rinse the urological catheters was proposed to cover their surface during application to prevent the formation of biofilms [58]. In addition, infections caused by *P. aeruginosa* biofilms were treated with a cocktail containing six lytic phages and tested with encouraging results [59]. Another cocktail combining six phages was tested to eradicate *P. aeruginosa* biofilms in a mice model with acute respiratory infection, showing great efficacy in disrupting biofilms [60]. Finally, two-phage cocktails are sometimes sufficient, as demonstrated to treat *E. faecalis* biofilms [61], although it is recommended to include more phages to reduce emergence of resistance.

Some phage-based products already on the market have been proposed as promising tools to remove biofilms [62], such a staphylococcal bacteriophage, containing the monophage *Sb-1*, which has been used in patients with osteomielytis and in foot ulcers [63], and *PYO* bacteriophage, a complex preparation designed for wound treatment [64]. There are also some commercially available phage-based products for the food industry against *Listeria sp*. [65,66] or *E. coli* [67] with bactericidal effects that are interesting for biofilm prevention. Some of these commercially available products, as Listex P100 [65] may also be promising for biofilm removal in surfaces of working environments of the food industry [68].

### 4.2. Phage-Derived Enzymes

**Figure 3:**
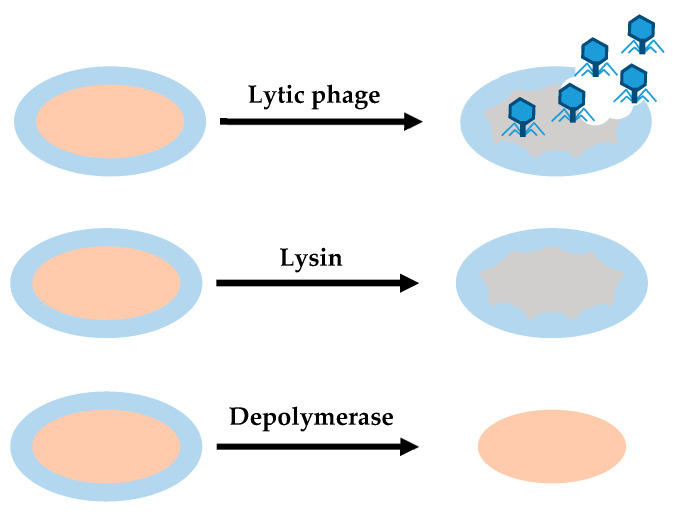
Differences between the action of lytic phages, lysins and depolymerases. Lytic phages provide antibacterial effect, degrading cell wall and EPS. Lysins provide a bactericidal effect, disrupting cell walls when they establish contact with their target. Depolymerases degrade EPS.

Some enzymes encoded with phages may be useful for treating bacterial infections and biofilms [69]. These enzymes or enzybiotics derived from phages can be used as an alternative to antibiotics for human and animal health. Their efficacy has been demonstrated in a few pre-clinical studies, but these products are still under development. Under current safety standards and regulations, the application of phage products is easier than use of the phage itself. According to this view, two main types of phage degradation enzymes are useful in the removal of biofilms: lysins and depolymerases (Figure 3).

#### 4.2.1. Lysins

Lysins are peptidoglycan hydrolases that have a bactericidal effect on susceptible bacteria. They break peptidoglycan bonds, degrading the bacterial cell wall and biofilm structure [70,71,72]. This makes lysins useful for Gram-positive bacteria [73]. Lysins are not restricted to be encoded by phages, since some bacteria produce lysins used to compete with other bacteria. In phages, lysins can be soluble enzymes, such as proteins that act at the end of the phage cycle to lysate the cell. In addition, they can be found in phage tails as virion-associated lysins, acting after receptor recognition to degrade the cell wall locally and allow injection of phage genomic material [74]. Depending on the peptidoglycan bonds they break, lysins are classified into different categories. Glycosidases or glycoside hydrolases break glycosidic bonds in complex sugars, N-acetylmuramoyl-L-alanine amidases cleaves the link between N-acetylmuramoyl residues and L-amino acid residues in certain cell-wall glycopeptides, and endopeptidases are proteolytic peptidases that break peptide bonds in non-terminal amino acids [75].

Phages that encode lysins have co-evolved with bacteria, so the binding domain of these enzymes evolved to target a unique and essential molecule in the cell wall, peptidoglycan, a well-preserved structure [71,76,77]. Lysins have been shown to exhibit thermostability, high ionic tolerance, and synergistic activity with antibiotics and other lysins [74]. In addition, lysins can be engineered to modify their target specificity and improve killing activity [78]. An example is the chimeric lysin Csl2, obtained by fusion of the catalytic domain of Cp1-7 lysozyme to the CW-7 repeats of the LySMP lysine from a *Staphylococcus suis* phage. It was designed to remove *S. suis* biofilms with positive results in vitro, and validated in vivo with a zebrafish infection model [79].

One of the main interesting features of lysins as therapeutic agents is that their activity is independent of the bacterial physiological state [79]. It was shown that the use of Art-175 lysine against multi-drug-resistant *P. aeruginosa* biofilms caused osmotic lysis independent of bacterial metabolism. This is relevant for biofilm removal because lysins can destroy persistent bacteria within biofilms, even at low metabolic rates [77].

#### 4.2.2. Depolymerases

Depolymerases are enzymes derived from phages that facilitate the early stages of phage infection by degrading the extracellular substances of encapsulated bacteria, and may also help to reach phage receptors [80]. They are capable of degrading the chains of capsular polysaccharides, exopolysaccharides, and O-polysaccharides from lipopolysaccharides and peptidoglycan. All these substances may constitute the capsule of some free-living bacteria, but most of them are important components of the biofilm matrix. Depolymerases can be associated with virions, forming part of the phage particle, or be in soluble form. The latter type of depolymerase can be released during lysis of the bacterial cell [74]. Due to the ability of phage-encoded depolymerases to degrade the polysaccharides in the bacterial capsule and biofilm matrix, phages encoding these enzymes may have easier access to the bacterial host, allowing infection. Therefore, depolymerase activity is particularly interesting in the removal of biofilms, as it alters the EPS matrix and decreases bacterial virulence [80].

Depolymerases are divided into different groups. Hydrolases are depolymerases that use one molecule of water to hydrolyze chemical bonds, while lyases catalyze the breaking of chemical bonds by means other than hydrolysis and oxidation. A third type of depolymerases are triacylglycerol lipases. They act on the carboxylic ester bonds of triacylglycerols by releasing organic acids and glycerol. In addition to the diversity of depolymerase general modes of action, within each category there is also a great diversity and depolymerases are highly target-specific. This diversity and specificity is a result of phage-host co-evolution, influenced by intense horizontal gene transfer [74,80]. Depolymerases are especially interesting for treating human or animal infections caused by biofilms. They can enhance the action of the immune system against bacteria by degrading the EPS matrix and allowing immune cells to access the bacteria in the biofilm [69].

Depolymerases have been tested against biofilms formed by different bacterial species. Depolymerase Dpo7, derived from the *vB_SepiS-phiPLA7* phage, was shown to reduce *Staphylococcus sp.* biofilm biomass by 53%–85% in 67% of the bacterial strains tested, in a dose-dependent but time-independent response [81]. Another example of depolymerase tested on biofilms with interesting results is Dpo42, derived from phage *vB_EcoM_ECOO78*. Its anti-biofilm activity was tested against *Escherichia coli*, again exhibiting dose-dependent biofilm prevention activity [82]. Finally, lysins and depolymerases are also good anti-biofilm agents in combination. For instance, lysin LysK and depolymerase DA7 have been tested in combination against *Staphylococcus aureus* biofilms in static and dynamic models. These enzymes showed a synergistic behavior, significantly reducing the number of viable cells in the biofilm [83].

### 4.3. Genetically Modified Phages

Penetration and diffusion of phages through the EPS-matrix is mandatory to eliminate biofilms using phage-based treatments. As mentioned above, some phage degradation enzymes serve this purpose, but many phages do not encode for these specific enzymes. However, phages can be genetically modified to produce enzymes that degrade the EPS-matrix, facilitating the removal of biofilms [84]. For example, a modified *T7 E. coli* phage has been designed to express intracellularly a hydrolase that is released during infection to the extracellular matrix, enhancing biofilm degradation. Testing on *E. coli* biofilms showed an elimination rate greater than 99%, and demonstrated the benefits of using manipulated phages [85].

Some temperate phages may have phenotypic characteristics that make them useful for biofilm removal. Genetic engineering can be used to turn these phages into lytic phages. This has been done by modifying the lysogenic *ΦEf11 E. faecalis* phage. *E. faecalis* biofilms are commonly associated with cases of failed root canals and nosocomial infections. *ΦEf11* was genetically modified to eliminate all genes related to lysogeny, eliminating transduction problems and achieving a significant reduction in the biomass of treated *E. faecalis* biofilms, both resistant and sensitive to the antibiotic vancomycin [51].

Another interesting feature of genetically modified phages is related to host range. In a recent study, researchers modified the genome of *T7Select E. coli* phage by inserting coding sequences for 1080, a short peptide with a broad-spectrum anti-biofilm effect. The modified phage was more effective in eradicating established *E. coli* biofilms than the unmodified phage [86]. Phages can also be designed to selectively kill antibiotic resistant bacteria. In addition, although lytic phages are typically used to destroy bacteria, temperate phages may be of interest for delivering programmable DNA nucleases associated with CRISPR to reverse antibiotic resistance. This system can selectively destroy plasmids that confer antibiotic resistance [87].

Lytic phages infect host cells in order to replicate and release new virions, leading to an exponential increase of viral populations along time. In addition, as replicating evolving entities, phages could potentially induce gene transduction and other drawbacks. In order to avoid these problems, phages could be modified. An interesting example is phage *AuNR,* genetically modified to express a receptor-specific binding protein to attach to several Gram-negative organisms. In addition, they were conjugated to gold nanorods, that following excitation by near-infrared light, induced the photothermal lysis of the targeted cells, also destroying the phages and avoiding replication. This phage treatment was tested over *P. aeruginosa* biofilms, showing widespread bacterial cell death even when they were cultured in mammalian epithelial cells. Thus, combination of gold nanorods and genetically modified phages results in an interesting tool for biofilm removal [88].

### 4.4. Phages in Combination with Antibiotics

A sub-lethal dose of antibiotics can stimulate phage virulence under certain conditions. This phenomenon is known as phage-antibiotic synergy (PAS). The idea of combining phage therapy and antibiotics comes from the understanding that by using two different selective pressures we can obtain more efficacy than by using each separately [41,89]. An example of the success of the combination of phages and antibiotics was demonstrated in a study in which the *Sb-1 S. aureus* phage increased antibiotic activity against biofilms. Phage *Sb-1* is particularly interesting for the treatment of *S. aureus* biofilms because of its ability to degrade the EPS-matrix [90]. Combination therapy of phages and antibiotics on *E. coli* biofilms has also been tested using *T4* phages and tobramycin, which strongly reduced antibiotic-resistant bacteria. The same test was done for *P. aeruginosa* biofilms, using phage *PB-1* [91].

The combination of phage-derived enzymes with antibiotics, such as the combination of depolymerases with antibiotics, can increase the antibacterial effect by facilitating the access of antibiotics to the bacteria within the biofilm [69]. In a study of bacterial biofilms in food processing environments, the action of a thermally stable depolymerase obtained from a *Klebsiella* phage was tested. The enzymatic pre-treatment increased the subsequent disinfection effect of chlorine dioxide, a broad-spectrum sterilizer commonly used in the food industry. This enzyme reduced the adhesion of bacteria and EPS-matrix, favoring the action of chlorine dioxide [92].

However, it is important to note that the combination of phages and antibiotics also has some drawbacks. This may lead to the emergence of double-resistant bacteria, similar to antibiotic cocktails [93]. In addition, phages may preferentially infect antibiotic-sensitive bacteria compared to those that form antibiotic-resistant biofilms, further promoting antibiotic resistance [77,93]. Moreover, antibiotics could potentially interfere with bacterial metabolism, which is required for phages to infect bacteria. For these reasons, the effects of double treatment of phages with antibiotics should be tested to avoid incompatibilities, as antagonistic effects could arise [94,95,96].

Designing phage cocktails that include antibiotics has also been considered. The use of phage cocktails and antibiotics is especially interesting for treating multiple bacterial infections because some pathogenic species or strains may be favored by eliminating competitors [97].

## 5. Problematic Biofilms in Anthropogenic Spaces

### 5.1. Infectious Diseases Caused by Biofilms

Infections caused by biofilms account for about 65% of all bacterial infections [98]. One of the most common infections caused by biofilms in humans is periodontitis, usually caused by poor oral hygiene, causing damage to the gums and teeth [2,8]. The bacterial species most frequently involved in periodontitis are *Pseudomonas aerobicus* and *F. nucleatum*, which form multi-species biofilms in the oral cavity and produce plaques that are mineralized with calcium and phosphate [2]. Phage therapy to treat periodontal disease has been tested in a few studies with encouraging results, including the use of whole virions, and also phage-coded enzymes that target several strains, which is especially important in order to fight multi-species biofilms, as preventive tools. These enzymes can be included in mouthwashes, toothpastes, or chewing gums. [99,100]. Another example of infectious diseases caused by biofilms is otitis media, which can be caused by various bacteria such as *Haemophilus influenzae*, *Moraxella catarrhalis*, and *S. aureus* among others [8,101]. These bacteria are also associated with chronic infections of the respiratory tract, where bacteria find an appropriate environment for establishment [101]. However, phage therapy has not yet been tested for these biofilm infections. Another global threat are biofilms identified in wounds, especially those that take 6 weeks or more to heal. Biofilms are associated with about 80% of surgical site infections and cause more complications in wound healing [102]. In vivo phage therapy has been assayed to treat these complications, especially against *E. coli* and *P. aeruginosa* [103], although the results obtained were inconclusive.

### 5.2. Biofilms in Medical Environments and Medical Devices

Biofilms are also important colonizers of hospital facilities and medical devices such as catheters, artificial implants, contact lenses, urinary prostheses, or orthotics, causing many device-associated infections [18]. For example, *P. aeruginosa* is one of the most important bacteria forming biofilms on contact lenses, causing keratitis [2,104]. Biofilms can also affect mechanical heart valves and surrounding tissues, causing prosthetic valve endocarditis. These biofilms are often established by *Streptoccocus sp*., *Bacillus sp*., and *Enterococcus sp*. [2]. Biofilms also play an important role in urinary tract infections, about 80% of which are associated with catheters [105]. The main species of biofilms on urinary catheters are *E. coli*, *Staphylococcus epidermis, E. faecalis, P. mirabilis, P. aeruginosa, Klebsiella pneumoniae*, and other Gram-negative species [106,107]. In a recent study, biofilm formation was analyzed in a total of 1070 urine samples from patients who showed at least two symptoms of urinary tract infection over two days or more. Biofilm formation was detected in 73.4% of the cases, demonstrating the high risk of catheter-associated urinary tract infections [108]. Phage therapy has been tested against some common urinary catheter biofilms, such as mixed biofilms of *P. aeruginosa* and *P. mirabilis* [109]. The proposed treatment, a cocktail of phages targeting both species (which is necessary in order to remove multi-species biofilms), achieved a significant decrease in bacterial populations of both species [97]. Another example are biofilms established in respirators, which cause ventilator-associated pneumonia mainly due to *P. aeruginosa* and *Acinetobacter baumannii* [101]. Phage therapy and phage lysins have already been proposed as possible agents for treating patients with ventilator-associated pneumonia [110]. In addition, phage therapy, alone or in combination with antibiotics, has been proposed for post-operative joint and bone infections, as biofilm formation in surface biomaterials, mainly by *S. aureus*, is a major concern [111].

### 5.3. Biofilms in Industrial Sectors

The formation of biofilms in the industry represents a major issue, particularly in the food industry. A large number of bacteria that cause food-borne diseases are capable of forming biofilms on most surfaces in food production plants, from where these pathogenic bacteria come into contact with and contaminate food. In food production plants, hygiene procedures are applied to avoid foodborne pathogens, but biofilms are very tolerant to these treatments [112,113]. An example of such foodborne pathogens forming biofilms is *Listeria monocytogenes*, which causes listeriosis [114]. *Clostridium perfringens* is also a biofilm-forming pathogen that causes food poisoning in humans and enterotoxemia in animals. This pathogen forms biofilms in livestock and poultry used for human consumption [115]. A few companies have developed phage-based treatments against *Listeria sp*. and *E. coli* to reduce the use of antibiotics, and can be used directly on food contact surfaces or in the processing environment [116], some of which are already available on the market as mentioned previously (Listex P100 [65], ListShield [66], and EcoShield [67]). These products are more anti-bacterial than anti-biofilm, and have been tested primarily as such. However, they would prevent the formation of new biofilms. Its commercialization shows the possible implementation in the near future of phage-based products specially designed for biofilm removal in the food industry. Noteworthy, Listex P100 has been tested for *L. monocytogenes* biofilm removal on steel surfaces, showing promising results and its possible use in working environments [68]. Another interesting example is the successful use of a phage cocktail against by *Staphylococcus lentus* and *Pseudomonas fluorescens*, two species that coexist in dairy plants forming dual-species biofilms [38], which could potentially prevent the formation of biofilms.

Water systems and pipes are amongst the industrial devices most affected by biofilms. Cooling water systems are usually colonized by biofilms. They can induce corrosion and damage to equipment, which is a significant economic loss. In addition, biofilms established in these systems may contain pathogenic bacteria. The predominant species in cooling water systems belong to the alpha-proteobacteria, beta-proteobacteria, and acidobacteria groups [117,118]. Biofilms are also a major source of contamination in drinking water distribution systems, causing water odor, corrosion of pipes, and potential health issues. Bacteria infecting drinking water systems include *Pseudomonas sp.*, *Janthinobacterium sp.,* and *Methilophilus sp*. Phage-based methods have been proposed to remove biofilms from the surfaces of drinking water systems, but further research is still needed in this area [119].

## 6. Conclusions

Biofilms are pervasive and form protected bacterial communities owing to an EPS-matrix that provides mechanical stability, adhesion to surfaces, and shielding from external dangers. Biofilms are very common in nature, but also in anthropogenic areas, where they represent a great challenge. It should be noted that biofilms can cause serious infections when they become established in the human body. Severe infections can also originate from biofilms established in medical devices such as catheters or mechanical heart valves. The industry is also affected by biofilms, especially the food industry and water distribution systems.

Phage-based therapies have become interesting alternatives for biofilm removal. These alternatives include the application of simple lytic phages, phage cocktails, phage derived enzymes, genetically manipulated phages, and also their use in combination with traditional antibiotics. The diversity of possible treatments is wide, allowing the design of the appropriate treatment for each case, and favoring personalized medicine. However, further research is still needed to improve efficacy and safety protocols, taking into account the particularities of each biofilm. In addition, more knowledge about phages is needed to increase social acceptance of phage-based treatments in order to improve their regulation and promote their use in the health and industrial areas.
